# Prevalence and Molecular Profiling of Merkel Cell Polyomavirus in Patients With Monkeypox Virus Infection

**DOI:** 10.1002/jmv.70890

**Published:** 2026-03-27

**Authors:** Sara Passerini, Davide Mariotti, Sara Messina, Valentina Mazzotta, Giulia Matusali, Andrea Antinori, Valeria Pietropaolo, Fabrizio Maggi, Luigi Rosa

**Affiliations:** ^1^ Department of Public Health and Infectious Diseases Sapienza University of Rome Rome Italy; ^2^ Laboratory of Virology and Laboratories of Biosecurity National Institute for Infectious Diseases Lazzaro Spallanzani – IRCCS Rome Italy; ^3^ Clinical Department National Institute for Infectious Diseases Lazzaro Spallanzani – IRCCS Rome Italy; ^4^ Department of Biology and Biotechnology “Charles Darwin” Sapienza University of Rome Rome Italy

**Keywords:** anal mucosa, HIV, MCPyV, MPXV

## Abstract

Mpox, caused by Monkeypox virus (MPXV), is associated with mucosal involvement and immune modulation that may influence viral coinfections. Merkel Cell Polyomavirus (MCPyV), a ubiquitous virus capable of lifelong persistence, was investigated in 66 Mpox patients enrolled at Lazzaro Spallanzani National Institute for Infectious Diseases (Rome, Italy; 2022–2025). Oropharyngeal and anal swabs collected during acute Mpox and, at 9‐month follow‐up, were analyzed by quantitative PCR, sequencing, transcript, and microRNA assays. MCPyV DNA was detected in 23/66 (34.8%) individuals, with higher prevalence and load in anal (31.8%, 2.1 × 10^3^ copies/mL) than in oropharyngeal swabs (24.4%, 1.3 × 10^2^ copies/mL; *p* < 0.001). MCPyV persisted in 4/10 (40%) oropharyngeal samples at follow‐up. No viral integration was observed, and full‐length Large Tumor Antigen was amplified in all samples. Transcript analysis revealed early and late genes; viral microRNAs were found in 3/10 (30%) oropharyngeal and 5/14 (35.7%) anal acute‐phase swabs, and persisted in 3/4 (75%) MCPyV‐positive oropharyngeal samples at follow‐up. Among the 12 MCPyV‐positive people living with human immunodeficiency virus (HIV), MCPyV load was lower in oropharyngeal but higher in anal swabs compared to MCPyV/MPXV cases. This study provides the first evidence of MCPyV detection in Mpox‐positive individuals and supports further investigation of its clinical relevance in coinfection settings.

## 1 Introduction

Recent Mpox outbreaks, caused by Monkeypox virus (MPXV), have raised concerns about coinfections with other viral pathogens and their potential clinical implications [1]. Previous data reported Mpox cases with concurrent viral infections [1], however, no studies addressed the prevalence of Human Polyomaviruses (HPyVs) among Mpox individuals. HPyVs are small DNA viruses that cause persistent and asymptomatic infections in healthy subjects. However, viral reactivation may occur in immunocompromised individuals, leading to serious diseases [2]. BK polyomavirus (BKPyV) can cause renal failure in kidney transplant patients, JC polyomavirus (JCPyV) triggers progressive multifocal leukoencephalopathy in AIDS patients and in individuals with autoimmune or hematological diseases under immunomodulatory therapy [2]. Among HPyVs, Merkel Cell Polyomavirus (MCPyV) is associated with a human tumor, the Merkel Cell Carcinoma (MCC), an aggressive skin cancer [3]. MCPyV double‐stranded DNA genome is divided into two coding regions separated by a Non‐Coding Control Region (NCCR) [4]. The early region produces the regulatory proteins, Large T (LT), small T (sT), and 57 kT antigens and the alternate LT open reading frame (ALTO), whereas the late one encodes for coat proteins, Viral Protein 1 and 2 (VP1, VP2) and for two mature microRNAs (miRNAs) that regulate LTAg levels [4]. MCPyV‐mediated oncogenesis involves clonal integration of viral DNA and the expression of a truncated LTAg that eliminates the ability of the virus to replicate [3, 4].

Despite its role in MCC, MCPyV usually establishes a lifelong persistence without pathological outcomes [5]. Viral DNA is frequently detected in the skin of healthy subjects, thus leading to consider MCPyV as a commensal of the skin microbiome [5].

The detection of MCPyV in the respiratory tract, digestive system, anal region, and other tissues indicates that this virus is widespread in the human body [6, 7]. However, immunodeficiency may induce viral reactivation and promote viral integration, thus contributing to carcinogenesis [5]. In this context, the interplay between MCPyV and other infections became of particular interest. A higher risk of developing MCC was reported in people living with HIV/AIDS (PLWH) [8]. Notably, acquired immunodeficiency and epithelial disruption associated with HIV may facilitate opportunistic infections, including HPyVs [8]. MCPyV DNA has been consistently detected in this population, particularly in the skin of men living with HIV [9], in sera of untreated individuals [10], and in anal swabs [11, 12, 13].

Moreover, MCPyV infection was associated with Human Papillomavirus (HPV) positivity in the anal tract [13]. The detection of MCPyV DNA in anal specimens from men who have sex with men (MSM) supports a potential sexual route of transmission, as also indicated by its frequent co‐detection with other sexually transmitted infections (STIs) [11, 12, 13, 14]. This hypothesis is further reinforced by the presence of concomitant STIs, such as *Chlamydia trachomatis* and *Mycoplasma genitalium* (*M. genitalium*), in the anal region of MCPyV‐positive individuals [14].

Within this framework, MPXV infection represents a biologically relevant but unexplored scenario due to the associated immune modulation, mucosal disruptions, and transmission by close contact.

Accordingly, this study was designed as an exploratory, hypothesis‐generating investigation aimed at assessing MCPyV prevalence, molecular state, viral transcripts, and miRNA expression in Mpox patients, as well as evaluating coinfections with other STIs, to provide preliminary insight into the biological state of MCPyV in Mpox individuals with established oncogenic risk factors.

## 2 Materials and Methods

A total of 66 patients with confirmed Mpox diagnosis were included. Oropharyngeal and anal swabs collected between May 2022 and May 2025 were analyzed.

Viral DNA was extracted with the QIAamp Viral RNA Mini Kit (Qiagen, Hilden, Germany). For the assessment of the viral load, a home‐made system was performed using an MPXV West African specific (G2R_WA) PCR assay [15] on the RotorGeneQ platform. Viral load was calculated from standard curves obtained from a ten‐fold dilution of a sample with known viral load (dilution range: 10^7^–10^2^ copies/mL). The RNAse P gene amplification was inserted as a human sample integrity/extraction control (Red Channel – TXR615).

MCPyV DNA was searched by quantitative polymerase chain reaction (qPCR) targeting the sT gene [13], and viral loads were calculated from standard curves obtained from ten‐fold plasmid dilutions (pMCV‐R17a, Addgene, #24729) (dilution range: 10^8^–10 copies/mL). MCPyV‐positive samples were further analyzed by PCR mapping NCCR and VP1 regions [12, 13], followed by sequencing (Bio‐Fab research, Rome, Italy). Alignment with the reference strain (GenBank Strain: EU375803) was performed using the Clustal W2 program. Viral integration sites were defined by the detection of the integrated papilloma sequence PCR technique, and LT truncation was examined as previously described [16].

In parallel, total RNA was extracted using the Quick‐RNA MicroPrep kit (Zymo Research Corporation, Irvine, CA, USA), reverse‐transcribed using the SensiFAST cDNA Synthesis kit (Meridian Bioscience, Cincinnati, OH, USA) and used for a PCR targeting MCPyV *LTAg* and *VP1* [16]. Moreover, to assess viral miRNA expression, the TaqMan miRNA assay for mcv‐miR‐M1‐5p (ID 006356) was employed, and RNU6B (ID001093) was used as an internal quality control.

All statistical analyses were conducted using GraphPad Prism 8 XML Project. Patient characteristics were summarized as median and interquartile range (IQR) for continuous and percentage for categorical variables. Comparisons of MCPyV loads were performed using the Mann–Whitney *U* test, and correlations between MPXV and MCPyV viral loads were assessed with Spearman's rank correlation. Statistical significance was set at *p* < 0.05.

## 3 Results

### 3.1 Demographics

The main characteristics of the study population are detailed in Table 1. A total of 66 self‐reported MSM patients were included in the study, with a median age of 42 years (IQR: 33–47). Nearly half of the cohort (48.5%) were PLWH, with a median CD4+ T‐cell count of 565 cells/μL (IQR: 429–870) recorded within the previous 3 months. Among PLWH, HIV viral load was undetectable in 34.4%, < 30 copies/mL in 53.1%, and > 30 copies/mL in 12.5% (ranging from 37 to 84 copies/mL). All people living with HIV included in the study were on stable antiretroviral therapy at the time of Mpox diagnosis.

**Table 1:** Main characteristics of the study population.

Demographic Mpox patients	
Male (*n*, %)	66 (100)
Age (median, IQR)	42 [33–47]
PLWH (*n*, %)	32 (48.5)
CD4 count in the past 3 months (median, IQR)	565 [429–870]
Undetectable viral load (*n*, %)	11 (34.4)
Viral load < 30 cp/mL (*n*, %)	17 (53.1)
Viral load > 30 cp/mL (*n*, %)	4 (12.5)
Concomitant STIs (*n*, %)	18 (27.3)
1 STI	12 (66.7)
2 STIs	5 (27.7)
3 STIs	1 (5.6)
Types of STIs (*n*, %)	
Gonorrhea	2 (5.1)
Syphilis	5 (12.8)
Mycoplasma^a^	7 (18.0)
Ureaplasma^b^	5 (12.8)
Anal HPV	6 (15.4)
MPXV sexual transmission route (*n*, %)	63 (95.5)
Systemic MPXV symptoms (*n*, %)	59 (89.4) yes/7 (10.6) no
History of smallpox vaccination (*n*, %)	5 (7.6) yes/52 (78.8) no/9 (13.6) unknown

*Note:* Percentages for STI types are calculated among patients with at least one STI and do not sum to 100%, as multiple infections could occur in the same individual.

Abbreviations: HPV, human papillomavirus; MPXV, Monkeypox virus; PLWH, people living with HIV; STIs, sexually transmitted infections.

^a^ 4/7 positive for *Mycoplasma hominis*, and 3/7 for *Mycoplasma genitalium*.

^b^ 5/5 positive for *Ureaplasma urealyticum*.

Concomitant STIs were reported in 27.3% of patients. Among these, 66.7% had a single STI, 27.7% had two STIs, and 5.6% had three STIs.

Systemic manifestations were observed in 89.4% of patients, while 10.6% exhibited only localized cutaneous lesions without systemic symptoms. Regarding prior smallpox vaccination status, 7.6% of individuals had a documented history of vaccination, 78.8% reported no vaccination, and in 13.6% the status was unknown.

### 3.2 Detection of MCPyV DNA in MPXV‐Positive Samples

MCPyV DNA was analyzed in 66 Mpox patients. Oropharyngeal swabs were obtained from 41 patients and anal swabs from 44 patients, with both sample types available for 18 patients. Oropharyngeal swabs were harvested during acute Mpox (T1) and follow‐up after 9 months of infection (T2), whereas anal swabs were collected only during the acute phase.

MCPyV DNA was detected in 23/66 (34.8%) Mpox patients. Specifically, viral DNA was found in 10/41 (24.4%) oropharyngeal and 14/44 (31.8%) anal swabs with a mean viral load of 1.3 × 10^2^ and 2.1 × 10^3^ copies/mL, respectively (Table 2).

**Table 2:** Prevalence and loads of MCPyV in oropharyngeal and anal swabs.

Sample	*n*	MCPyV+	MCPyV load (copies/mL)
Oropharyngeal swabs	41	10 (24.4%)	1.3 × 10^2^
Anal swabs	44	14 (31.8%)	2.1 × 10^3^
Nonlesional	32	9 (28.1%)	2.6 × 10^3^
Lesional	12	5 (41.6%)	1.15 × 10^3^
Total	85	24 (28.2%)	1.26 × 10^3^

Abbreviations: MCPyV, Merkel Cell Polyomavirus; mL, milliliters.

Of MCPyV‐positive oropharyngeal samples during the acute Mpox phase (T1), 4/10 (40%) remained positive at follow‐up (T2) with a mean viral load of 1.16 × 10^2^ copies/mL. At T2, MPXV DNA was not detected in any oropharyngeal sample.

**Figure 1:**
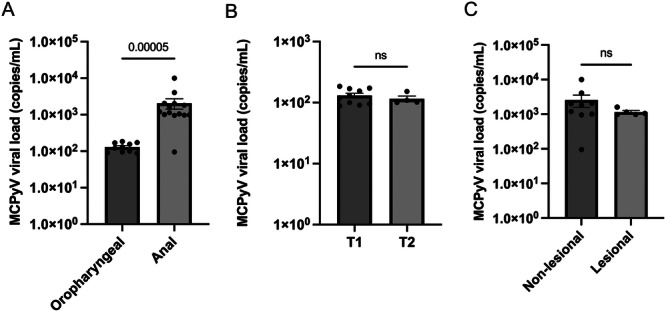
Merkel Cell Polyomavirus (MCPyV) load (A) in oropharyngeal and anal swabs, (B) among oropharyngeal swabs during acute Mpox (T1) and at follow‐up after 9 months of infection (T2), (C) among nonlesional and lesional anal swabs. Each dot represents the MCPyV viral load of an individual patient, mean values ± standard error of the mean (SEM) are also shown. Comparisons between groups were performed using the Mann–Whitney *U* test. Statistical significance was defined as *p* < 0.05. ns, not significant.

Whereas no significant differences (*p* > 0.05) were found between oropharyngeal swabs at T1 and T2 (Figure 1B), nor among nonlesional and lesional anal swabs (Figure 1C).

Among anal samples, MCPyV was detected in 9/32 (28.1%) nonlesional and 5/12 (41.6%) lesional swabs with a mean viral load of 2.6 × 10^3^ and 1.1 × 10^3^ copies/mL, respectively (Table 2). Viral loads were significantly higher in anal than in oropharyngeal swabs, corresponding to an approximately 16‐fold difference (*p* = 0.00005) (Figure 1A), whereas no significant differences were found between oropharyngeal swabs at T1 and T2, corresponding to an approximately 1.1‐fold difference (*p* > 0.05) (Figure 1B), nor between nonlesional and lesional anal swabs, despite an approximately 2.4‐fold higher mean viral load in nonlesional samples (Figure 1C).

In a subset analysis of 18 patients for whom both oropharyngeal and anal swabs were available, 11 patients showed concordant MCPyV results (10/11 negative and 1/11 positive in both swabs), whereas the remaining 7 patients showed discordant MCPyV results (6/7 positive only in the anal and 1/7 positive only in the oropharyngeal swabs).

To further explore the potential relationship between the two viruses, a correlation analysis was performed between MCPyV and MPXV viral loads, revealing a negative but nonsignificant association (Spearman's *ρ* = −0.307, *p* = 0.200; 95% CI: −0.668 to 0.171) (Figure S1).

### 3.3 Sequencing of MCPyV Regions and Integration Sites Analysis

All MCPyV‐positive samples revealed canonical NCCR and VP1 structures. Moreover, no viral integration was observed, and a full‐length *LTAg* was amplified in all examined specimens.

### 3.4 Detection of Viral Transcripts and miRNAs

Transcript analysis revealed *LTAg* and *VP1* gene expression in all virus‐positive samples. Viral miRNAs were found in 3/10 (30%) oropharyngeal and 5/14 (35.7%) anal MCPyV‐positive swabs collected during the acute phase. At T2, mcv‐miR‐5p was detected in 3/4 (75%) MCPyV DNA‐positive oropharyngeal samples.

### 3.5 Coinfection Analysis

Among MCPyV‐positive individuals, 12 were PLWH (Table 3). In oropharyngeal swabs, MCPyV load was modestly lower in MCPyV/HIV/Mpox than in MCPyV/Mpox patients (approximately 1.2‐fold difference), whereas the opposite pattern was observed in anal swabs, where MCPyV load was approximately 1.6‐fold higher in MCPyV/HIV/Mpox coinfected patients (Figure S2A,B). In addition, other coinfections, including Syphilis (1), *Mycoplasma hominis* (3), *M. genitalium* (2), *Ureaplasma urealyticum* (1), and anal HPV (2) were reported (Table 3).

**Table 3:** Coinfections in Mpox cases according to MCPyV status.

Concomitant coinfections	MCPyV+, *n*	MCPyV−, *n*
HIV	12	20
Syphilis	1	4
*Mycoplasma hominis*	3	1
*Mycoplasma genitalium*	2	1
*Ureaplasma urealyticum*	1	4
Anal HPV	2	4

Abbreviations: HIV, human immunodeficiency virus; HPV, human papillomavirus; MCPyV, Merkel Cell Polyomavirus; STIs, sexually transmitted infections.

## 4 Discussion

Viral coinfections represent an important challenge in infectious diseases, as they may influence clinical severity, treatment response, and long‐term outcomes [1, 17]. In emerging viral infections such as Mpox, investigating concomitant viruses may improve understanding of disease pathogenesis. HPyVs, characterized by lifelong persistence, reactivation in immunocompromised hosts, and oncogenic potential [2], have not been previously studied in the context of MPXV coinfection. This study provides the first evidence on MCPyV prevalence and molecular characteristics in Mpox patients. Although the study is primarily descriptive, the combined detection of MCPyV DNA, viral transcripts, miRNAs, and long‐term persistence provides preliminary descriptive insight into the biological state of MCPyV during acute and convalescent MPXV infection, suggesting active regulation rather than passive carriage.

MCPyV DNA was detected in about 35% of patients, with a higher prevalence and viral load in anal swabs compared to oropharyngeal samples. This finding is consistent with previous reports highlighting the frequent detection of MCPyV in the anal tract of MSM populations, thus supporting the anal region as a preferential site for MCPyV tropism and the plausible sexual transmission of the virus [11, 12, 13, 14]. In this framework, the choice of rectal and oropharyngeal swabs was driven by the study aim of exploring MCPyV in a Mpox‐positive MSM population, in which these mucosal compartments are directly involved in sexual exposure and viral transmission. Sampling these sites therefore allows the assessment of viral presence in tissues of direct epidemiological relevance. Moreover, mucosal sampling may better capture asymptomatic or subclinical viral shedding, providing insights into viral persistence and dissemination.

In line with this, differences in local mucosal immunity may contribute to the observed site‐specific distribution, as infections at nongenital sites may be less prone to reactivation from latency compared with genital compartments, where microtraumas and concomitant STIs may impair local viral clearance [18]. No clear association was observed between MCPyV positivity and clinical severity indicators of Mpox, including lesion burden, systemic symptoms, or duration of MPXV detection, suggesting that MCPyV presence may reflect background viral persistence rather than disease severity.

In line with previous studies describing persistent MCPyV replication in different anatomical sites [6], the detection of viral transcripts, as well as the absence of integration events, truncated LTAg, or relevant sequence variability in NCCR and VP1 regions, confirms the commensal role of MCPyV in the skin and mucosa of immunocompetent individuals [5]. As JCPyV and BKPyV, also MCPyV encodes for viral miRNAs capable to target *LTAg* transcripts and modulate MCPyV replication thus promoting episomal persistence [19, 20]. Therefore, the isolation of mcv‐miR‐5p may support the establishment of a persistent infection. Notably, MCPyV DNA and miRNAs were still detectable in a subset of oropharyngeal swabs at 9 months after acute MPXV infection, suggesting the possibility of long‐term persistence [19, 20] and emphasizing the importance of follow‐up investigations.

An interesting finding was that among the 18 patients with both oropharyngeal and anal swabs available, only 11 showed concordant MCPyV detection, while 7 exhibited viral DNA in a single site (6 anal swabs; 1 oropharyngeal swab). This observation indicates site‐specific variability in MCPyV detection [6] and highlights the importance of multisite sampling in coinfection studies to avoid underestimation of viral prevalence.

Correlation analysis between MPXV and MCPyV loads showed a negative trend that did not reach statistical significance. Therefore, no association between the two viruses can be inferred from these data. However, this observation was considered hypothesis‐generating, as immune activation during acute viral infections is known to influence the persistence and expression of commensal or latent viruses, including HPyVs [21]. Larger longitudinal studies will be required to determine whether any interaction between MPXV and MCPyV occurs in vivo.

Consistent with previous studies [13, 14], the co‐occurrence of MCPyV with other STIs, particularly HIV, was reported, emphasizing host immunosuppression as critical for MCPyV infection. Interestingly, opposite trends of MCPyV load were observed depending on the anatomical site, reflecting distinct local immune environments or a synergistic effect of MPXV‐induced inflammation and HIV‐related immunosuppression.

Despite the integration of virological and clinical data provides novel insight into the interplay between MCPyV and MPXV, this study is mainly observational and hypothesis‐generating and has some limitations. The absence of an Mpox‐negative MSM control group limits the ability to determine whether MCPyV prevalence differs from that in comparable populations. Longitudinal data were limited to a small number of oropharyngeal samples, with no follow‐up anal specimens, limiting assessment of site‐specific viral persistence and evaluation of oncogenic outcomes. The lack of histological data precludes any direct inference on the potential role of MCPyV in Mpox‐associated lesions. Given the exploratory nature of the study and the outbreak‐related setting, no a priori power calculation was performed, and the relatively small sample size may have limited the statistical power of correlation analyses and highlights the need for further research.

In conclusion, this study revealed that MCPyV is detectable in a substantial proportion of patients with Mpox, particularly in anal samples, and that viral persistence can occur during and after acute infection. Although no evidence of oncogenic integration, truncation, or significant viral load correlation was observed, these findings provide baseline data on MCPyV presence and molecular state in the context of Mpox. This work offers baseline evidence for future studies, including larger, multicenter, and long‐term studies, aimed at clarifying the clinical relevance of MCPyV detection, its transmission dynamics, and its potential role in long‐term outcomes.

## Author Contributions

**Sara Passerini:** conceptualization, investigation, data curation, writing – original draft preparation, writing – review and editing. **Davide Mariotti:** investigation, data curation, writing – original draft preparation, writing – review and editing. **Sara Messina:** investigation, data curation. **Valentina Mazzotta:** supervision, writing – review and editing. **Giulia Matusali:** conceptualization, writing – review and editing. **Andrea Antinori:** supervision, writing – review and editing. **Valeria Pietropaolo:** conceptualization, supervision, funding acquisition, writing – original draft preparation, writing – review and editing. **Fabrizio Maggi:** supervision, funding acquisition, writing – review and editing. **Luigi Rosa:** conceptualization, investigation, data curation, writing – original draft preparation, writing – review and editing.

## Ethics Statement

The study was conducted at the Lazzaro Spallanzani National Institute for Infectious Diseases IRCCS (Rome, Italy) under the “Mpox‐Cohort” protocol. The study was approved by the Ethical Committee of the Lazzaro Spallanzani Institute (MpoxCohort protocol: “Studio di coorte osservazionale monocentrica su soggetti che afferiscono per sospetto clinico o epidemiologico di malattia del vaiolo delle scimmie (mpox)”; Approval Number 40z, Register of Non‐Covid Trials 2022).

## Consent

All participants provided written informed consent.

## Conflicts of Interest

The authors declare no conflicts of interest.

## Supporting information


**Figure S1:** Correlation analysis between Merkel Cell Polyomavirus (MCPyV) and Monkeypox virus (MPXV) loads using Spearman's rank correlation test. 95% confidence interval (CI) is also reported by dotted blue lines. Statistical significance was defined as p < 0.05. **Figure S2:** Merkel Cell Polyomavirus (MCPyV) load in (A) oropharyngeal swabs and (B) anal swabs from co‐infected patients. Each dot represents the MCPyV load of an individual patient; mean values ± standard error of the mean (SEM) are also shown. Comparisons between groups were performed using the Mann‐Whitney *U* test. Statistical significance was defined as *p* < 0.05. ns: not significant.

## Data Availability

The data that support the findings of this study are available from the corresponding authors upon reasonable request.
