# Supplementary material for: Prevalence and Molecular Profiling of Merkel Cell Polyomavirus in Patients With Monkeypox Virus Infection
Source: J Med Virol. 2026 Mar 27;98(4):e70890. doi: 10.1002/jmv.70890 (PMC13023021; doi:10.1002/jmv.70890)
Supplement: Supplementary file 1 — Figure S1: Correlation analysis between Merkel Cell Polyomavirus (MCPyV) and Monkeypox virus (MPXV) loads using Spearman's rank correlation test. 95% confidence interval (CI) is also reported by dotted blue lines. Statistical significance was defined as p < 0.05. Figure S2: Merkel Cell Polyomavirus (MCPyV) load in (A) oropharyngeal swabs and (B) anal swabs from co‐infected patients. Each dot represents the MCPyV load of an individual patient; mean values ± standard error of the mean (SEM) are also shown. Comparisons between groups were performed using the Mann‐Whitney U test. Statistical significance was defined as p < 0.05. ns: not significant. [file JMV-98-e70890-s001.docx]

**Supporting Information**

**Materials and Methods**

**Study design, participants and sample collection**

Patients with confirmed Mpox diagnosis, admitted at the Lazzaro Spallanzani National Institute for Infectious Diseases IRCCS (Rome, Italy) from 2022 to 2025, were enrolled in the ‘Mpox-Cohort’ protocol and followed up to 1 year from diagnosis. All patients provided written informed consent to participate in the study. The study was approved by the Ethical Committee of the Lazzaro Spallanzani Institute (MpoxCohort protocol: *“Studio di coorte osservazionale monocentrica su soggetti che afferiscono per sospetto clinico o epidemiologico di malattia del vaiolo delle scimmie (mpox)”;* approval number 40z, Register of Non-Covid Trials 2022). All MPXV infections included in this study belonged to clade II. We included in this study 66 people with oropharyngeal and anal swabs available for analysis from May 2022 up to May 2025.

Oropharyngeal swabs were collected during the acute phase of MPXV infection (T1) and, when available, at follow-up 9 months after diagnosis (T2). Anal swabs were collected only during the acute phase (T1), as patients declined repeat rectal sampling at follow-up due to the invasive nature of the procedure in the absence of symptoms. In addition, previous longitudinal studies have shown that MPXV DNA in rectal samples typically becomes undetectable within 4-6 weeks after symptom onset, supporting the decision to restrict anal sampling to the acute phase [1]. In order to prevent degradation, both oropharyngeal and anal swabs were stored at -80°C until use for DNA and RNA extraction.

**DNA extraction and MPXV load**

Viral DNA was extracted with the QIAamp Viral RNA Mini Kit (Qiagen, Hilden, Germany). For the assessment of the viral load, a home-made system was performed using an MPXV West African specific (G2R_WA) PCR assay [2] on the RotorGeneQ platform. The target viral gene of the PCR assay is the G2R; Primer sequences are the following: Forward primer (5′-CACACCGTCTCTTCCACAGA); Reverse primer (5′-GATACAGGTTAATTTCCACATCG). PCR amplification was performed with an initial denaturation step at 95 °C for 2 min, followed by 45 cycles of denaturation at 95 °C for 15 s and annealing/extension at 59 °C for 60 s. Viral load was calculated from standard curves obtained from a ten-fold dilution of a sample with known viral load (dilution range: 10^7^-10^2^ copies/ml). The RNAse P gene amplification was inserted as a human sample integrity/extraction control (Red channel - TXR615).

**Detection of MCPyV DNA**

MCPyV DNA was measured by quantitative polymerase chain reaction (qPCR) using primers and probe targeting the sT gene [3]. Viral load was calculated from standard curves obtained from a ten-fold dilution of a plasmid containing the entire MCPyV genome (pMCV-R17a, Addgene, #24729) (dilution range: 10^8^-10 copies/ml).

**Detection and sequencing of MCPyV NCCR and VP1 regions**

MCPyV-positive samples were further analyzed by PCR mapping NCCR and VP1 regions [3,4], followed by sequencing (Bio-Fab research, Rome, Italy). Alignment with the reference strain (GenBank strain: EU375803) was performed using the Clustal W2 program.

**Analysis of integration sites and sequencing of LTAg**

Sample positive for MCPyV were also examined for viral integration [5]. The integration sites were defined by submitting sequences to the databases of the National Centre for Biotechnology Information and analyzing them with the Basic Local Alignment Search Tool (BLAST) for genomic localization. Moreover, to examine whether a truncated LTAg was expressed, a PCR was carried out using a combination of six primer sets [5] and a direct sequencing was carried out on the amplified products.

**RNA extraction and analysis of LTAg and VP1 genes**

Total RNA was extracted using Quick-RNA MicroPrep kit (Zymo Research Corporation, Irvine, CA, USA), according to the manufacturer’s instructions. DNase treatment was performed during RNA extraction to avoid DNA contamination in transcript analyses. Therefore, an aliquot of RNA was reverse-transcribed using the SensiFAST cDNA Synthesis kit (Meridian Bioscience, Cincinnati, OH, USA) and used for a PCR targeting MCPyV *LTAg* and *VP1* [5].

**Detection of viral miRNAs**

To assess viral miRNA expression, the pre-designed TaqMan microRNA assay for mcv-miR-M1-5p (ID 006356) was employed to detect MCPyV-encoded miRNAs, whereas RNU6B (ID001093) was used as an internal quality control.

**Statistical Analysis**

All statistical analyses were conducted using GraphPad Prism 8 XML ProjecT. Demographic and epidemiological characteristics of the patients were described using the median and Interquartile Range (IQR) for continuous parameters and absolute and relative (percentage) frequencies for categorical variables. Comparisons of MCPyV DNA loads were performed using the Mann-Whitney U test with Bonferroni correction, and correlations between MPXV and MCPyV viral loads were assessed with Spearman’s rank correlation and 95% confidence interval (CI). Statistical significance was set at p<0.05. Given the exploratory nature of the study and the absence of prior data on MCPyV-MPXV co-infection, no a priori power calculation was performed.

**Supporting Figures**

**
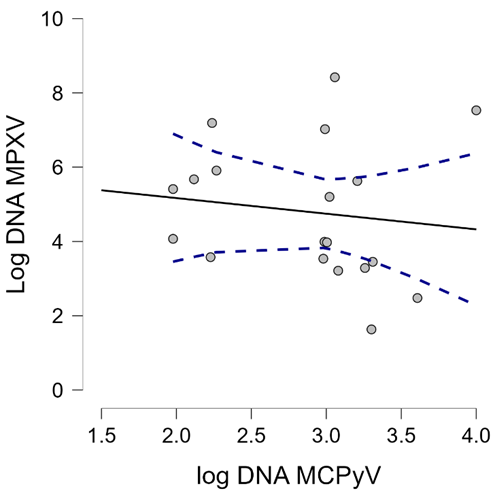
**

**Figure S1.** Correlation analysis between Merkel Cell Polyomavirus (MCPyV) and Monkeypox virus (MPXV) loads using Spearman’s rank correlation test. 95% confidence interval (CI) is also reported by dotted blue lines. Statistical significance was defined as p<0.05.

**
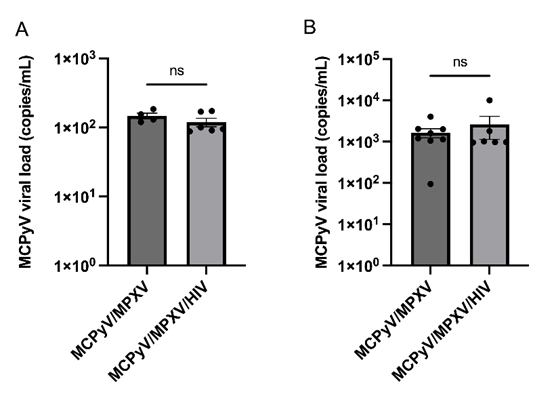
**

**Figure S2**. Merkel Cell Polyomavirus (MCPyV) load in (A) oropharyngeal swabs and (B) anal swabs from co-infected patients. Each dot represents the MCPyV load of an individual patient; mean values ± standard error of the mean (SEM) are also shown. Comparisons between groups were performed using the Mann-Whitney U test. Statistical significance was defined as p<0.05. ns: not significant.

**REFERENCES**

1. Suñer, C., Ubals, M., Tarín-Vicente, E. J., Mendoza, A., Alemany, A., Hernández-Rodríguez, Á., Casañ, C., Descalzo, V., Ouchi, D., Marc, A., Rivero, À., Coll, P., Oller, X., Miguel Cabrera, J., Vall-Mayans, M., Dolores Folgueira, M., Ángeles Melendez, M., Agud-Dios, M., Gil-Cruz, E., Paris de Leon, A., … Mitjà, O. Viral dynamics in patients with monkeypox infection: a prospective cohort study in Spain. Lancet. Infect. dis. 2023, 23(4), 445–453. doi:10.1016/S1473-3099(22)00794-0
2. Li, Y., Zhao, H., Wilkins, K., Hughes, C., & Damon, I. K. Real-time PCR assays for the specific detection of monkeypox virus West African and Congo Basin strain DNA. Journal of virological methods, 2010, 169(1), 223-227. doi: 10.1016/j.jviromet.2010.07.012.
3. Passerini S, Fracella M, Benvenuto D, Bugani G, D'Auria A, Coratti E, Babini G, Moens U, Cavallari EN, Torti C, Antonelli G, Ciccozzi M, Pierangeli A, d'Ettorre G, Scagnolari C, Pietropaolo V. High rates of anal Merkel Cell Polyomavirus and HPV co-infection among people living with HIV. J Med Virol. 2024 Aug;96(8):e29883. doi: 10.1002/jmv.29883.
4. Prezioso C, Obregon F, Ambroselli D, Petrolo S, Checconi P, Rodio DM, Coppola L, Nardi A, Vito C, Sarmati L, Andreoni M, Palamara AT, Ciotti M, Pietropaolo V. Merkel Cell Polyomavirus (MCPyV) in the Context of Immunosuppression: Genetic Analysis of Noncoding Control Region (NCCR) Variability among a HIV-1-Positive Population. Viruses. 2020 May 4;12(5):507. doi: 10.3390/v12050507.
5. Hashida Y, Imajoh M, Nemoto Y, Kamioka M, Taniguchi A, Taguchi T, Kume M, Orihashi K, Daibata M. Detection of Merkel cell polyomavirus with a tumour-specific signature in non-small cell lung cancer. Br J Cancer. 2013 Feb 19;108(3):629-37. doi: 10.1038/bjc.2012.567.
